# Semi-Poisson Statistics in Relativistic Quantum Billiards with Shapes of Rectangles

**DOI:** 10.3390/e25050762

**Published:** 2023-05-06

**Authors:** Barbara Dietz

**Affiliations:** Center for Theoretical Physics of Complex Systems, Institute for Basic Science (IBS), Daejeon 34126, Republic of Korea; barbara@ibs.re.kr

**Keywords:** quantum chaos, relativistic quantum chaos, quantum billiards, relativistic quantum billiard

## Abstract

Rectangular billiards have two mirror symmetries with respect to perpendicular axes and a twofold (fourfold) rotational symmetry for differing (equal) side lengths. The eigenstates of rectangular neutrino billiards (NBs), which consist of a spin-1/2 particle confined through boundary conditions to a planar domain, can be classified according to their transformation properties under rotation by π (π/2) but not under reflection at mirror-symmetry axes. We analyze the properties of these symmetry-projected eigenstates and of the corresponding symmetry-reduced NBs which are obtained by cutting them along their diagonal, yielding right-triangle NBs. Independently of the ratio of their side lengths, the spectral properties of the symmetry-projected eigenstates of the rectangular NBs follow semi-Poisson statistics, whereas those of the complete eigenvalue sequence exhibit Poissonian statistics. Thus, in distinction to their nonrelativistic counterpart, they behave like typical quantum systems with an integrable classical limit whose eigenstates are non-degenerate and have alternating symmetry properties with increasing state number. In addition, we found out that for right triangles which exhibit semi-Poisson statistics in the nonrelativistic limit, the spectral properties of the corresponding ultrarelativistic NB follow quarter-Poisson statistics. Furthermore, we analyzed wave-function properties and discovered for the right-triangle NBs the same scarred wave functions as for the nonrelativistic ones.

## 1. Introduction

This paper is a contribution to the special issue *Quantum Chaos*, which is dedicated to the 80th birthday of Giulio Casati who is a leading expert in the fields of classical and quantum chaos. He, actually, already expressed in [1] the conjecture that the spectral properties of quantum systems with a chaotic classical dynamics coincide with those of random matrices from the Gaussian ensembles (GEs) of corresponding universality class [2], that is, before Bohigas, Gianoni and Schmit formulated the famous BGS conjecture [3]. According to the BGS conjecture, they are well described by random marix theory (RMT) [2,4,5,6,7], where the appropriate GE for quantum systems with preserved and violated time-reversal invariance are the Gaussian orthogonal ensemble (GOE) and the Gaussian unitary ensemble (GUE), respectively. Criteria for its validity were identified in Ref. [8] based on the semiclassical periodic orbit (PO) theory, which was pioneered by Gutzwiller [9,10]. Casati considered a billiard with the shape of a stadium. Billiards provide a particularly suited model for studies in the context of quantum chaos. The dynamics of classical billiards (CBs), consisting of a point particle which moves freely inside a bounded two-dimensional domain and is reflected specularly at the boundary, can be engineered through the choice of their shape [11,12,13]. The eigenstates of the corresponding nonrelativistic quantum billiard (QB) are determined by solving the Schrödinger equation for a free particle and imposing the Dirichlet boundary condition (BC) on the resulting wave functions. Berry and Tabor showed in [14] based on action-angle variables that the spectral properties of typical integrable systems [15] agree well with those of Poissonian random numbers. However, there are numerous examples for ’untypical’ integrable systems, a paradigm one being the harmonic oscillator [16]. Another example is polygonal billiards [17,18,19], such as triangular billiards, which have been investigated by Casati and his collaborators for nearly three decades [20,21,22,23] and the rectangular billiard [24]. Billiards with the shapes of rational polygonals of which the boundary comprises diffractive corners with angles $\alpha_{i}=\frac{n_{i}}{m_{i}}\pi$ where $m_{i},n_{i}$ are integers and $n_{i}\neq 1$ [17,25,26,27,28,29,30,31,32] are neither integrable nor chaotic. Therefore, they are referred to as pseudointegrable systems. Their phase space trajectories propagate on invariant surfaces that are topologically equivalent to multihandled, two-dimensional tori with genus 2≤g<∞, implying that they are not ergodic in phase space. Yet, the motion in such billiards has a chaotic component arising from the diffractive corners, which are of measure zero in classical phase space. The spectral properties of the corresponding QB differ considerably from those of typical integrable systems [26,28,29,30,31]. To be more explicit, they exhibit features that are intermediate between those of Poissonian random numbers and those of random matrices from the GOE in the sense that the levels repel each other linearly like in chaotic systems, and their nearest-neighbor spacing distribution decreases exponentially for large spacings, which is typical for integrable systems [33]. Depending on the choice of angles of a right-triangle QB, the properties are well described by those of semi-Poissonian numbers, which are obtained by deleting every second one in a sequence of Poissonian ones [23,34]. Such cases are considered in the present work.

We present results obtained for the spectral properties, momentum distributions [35] and Husimi functions [36,37] of the rectangular and square-shaped relativistic neutrino billiards (NBs) and their symmetry-projected eigenstates. These are obtained by separating their eigenstates according to their transformation properties under rotation by π and π/2, respectively. Neutrino billiards were introduced by Berry and Mondragon [38]. They are governed by the Weyl equation [39] for a non-interacting, massless spin-1/2 particle—commonly referred to as the Dirac equation in this context [38]—with the BC that the outward current vanishes. In distinction to QBs, NBs and the relativistic quantum systems considered in Ref. [40] do not have a well-defined classical counterpart. Insight into their behavior in the semiclassical limit can be obtained based on a semiclassical approximation for the fluctuating part of their spectral density in terms of a trace formula [41,42], which is applicable from the ultrarelativistic limit for massless neutrinos to the nonrelativistic limit of large mass $m_{0}$ where the energy is close to the rest energy $E_{0}=m_{0}c^{2}$ [43]. The trace formula is a sum over periodic orbits of the CB of corresponding shape, where in the ultrarelativistic limit, those with an odd number of reflections at the boundary are missing.

An alternative type of billiards exhibiting relativistic features in part of their eigenvalue spectrum are graphene billiards, which are constructed by cutting a honeycomb lattice out of their shape. They are used to model properties of artificial graphene flakes based on a tight-binding model [44,45]. Finite-size sheets of graphene [46,47,48,49], referred to as graphene quantum dots, have the advantage that they can be studied experimentally. The first experiments were presented in [50,51,52]. In the vicinity of the corners of the hexagonal Brillouin zone, where the conduction and valence band touch each other conically, the energy excitations are governed by the Dirac equation for massless spin-1/2 particles [53]. The occurrence of the conical structure solely originates from the honeycomb structure of graphene, which is formed by two interpenetrating triangular lattices. This led to the realization of numerous experimental ’artificial-graphene’ realizations [54]. Boundary conditions on the spinor components in a graphene billiard were formulated in [55,56,57]. We modeled rectangular, Africa-shaped and threefold-symmetric graphene billiards experimentally with flat superconducting microwave photonic crystals [58,59,60,61,62] and found agreement with the spectral properties of massive neutrino billiards only beyond a certain mass. In addition, theoretical studies of rectangular graphene quantum dots yielded deviations from those of massless neutrino billiards [63]. Their origin is explained in Ref. [64] and may be attributed to the presence of the boundary and differing BCs. The extraordinary features presented in this work are only observed in the ultrarelativistic limit for massless neutrino billiards. Similar studies will be performed for graphene billiards for varying BCs in a separate work.

The work was motivated by results obtained in [65] for the equilateral triangle and in [66] for sectors of the circle and ellipse NB. The boundary of the equilateral triangle has a $C_{3v}$ symmetry [67], that is, threefold rotational symmetry and mirror symmetries with respect to its main axes: that of the circle belongs to the U(1) symmetry class, which comprises all *M*-fold rotational symmetries with M≥2, and that of the ellipse has mirror symmetries with respect to its minor and major axes and a twofold rotational symmetry. Generally, the eigenfunctions of a QB with a mirror symmetry are separated into eigenfunctions which are either symmetric or antisymmetric with respect to the symmetry axes, and they fulfill either Neumann or Dirichlet BCs along them. This is not possible for NBs. However, as will be outlined in Section 2, the eigenstates of NBs whose boundary has a *M*-fold rotational symmetry can be separated according to their transformation properties under rotation by $\frac{2\pi }{M}$ into symmetry-projected eigenstates. Sectors of NBs are constructed by cutting these along the borders of a fundamental domain associated with a discrete rotational symmetry and imposing the same boundary conditions along the cutlines as along the outer boundary, that is, as for the full NB. These NBs are referred to as symmetry-reduced NBs in the following. The circle and ellipse sector CBs have an integrable classical dynamic [68,69], and the corresponding QBs exhibit Poissonian statistics. The symmetry-projected eigenstates of the circle and ellipse NBs have been determined analytically in Refs. [38,41,65,70]. Their spectral properties also exhibit Poissonian statistics, that is, they agree with those of the corresponding QB. The spinor components of the eigenfunctions of an NB are linked through the BCs at the boundary [38]. Furthermore, for NBs with a discrete rotational symmetry, they transform differently under the associated rotation [65,71], implying that the symmetry classes are intermingled when cutting an NB into symmetry-reduced NBs. We demonstrated in [66] that consequently, the symmetry-reduced and full NBs cannot have any common eigenstates and that the spectral properties of symmetry-reduced circle NBs with an arbitrary inner angle smaller than 2π and the quarter-ellipse NBs with sufficiently small eccentricity agree with GOE after extracting the contributions from librational modes. This was also attributed to the discontinuity of the BC at the corners, where straight and curved parts are connected.

In [65], we computed the eigenstates of massive equilateral triangle NBs and their symmetry-projected eigenstates analytically and found that they coincide with those of the corresponding QB. Their short-range correlations exhibit the nontypical behavior expected for rectangular billiards whose side lengths are commensurable; however, otherwise, the spectral properties agree with semi-Poisson statistics. In contrast, those of the massless right-triangle NB, which is obtained by cutting the equilateral triangle NB along a mirror-symmetry axis, agree with Poisson [66]. These results are in contrast to those for the ellipse and circle NBs and corresponding sector NBs. They are attributed to the fact that the equilateral triangle NB has no curved boundary parts. In the present work, we investigate properties of the symmetry-projected and symmetry-reduced eigenstates of rectangular billiards with commensurable and incommensurable side length. The central question was whether the symmetry-projected eigenstates of rectangular NBs show a similar behavior as those of the equilateral triangle.

## 2. Review of Characteristic Features of Neutrino Billiards

In the two-dimensional plane r=(x,y), the Dirac equation for a free spin-1/2 particle with mass $m_{0}$ and momentum $\hat{p}=-i\hbar \nabla$ reads

(1) $${\hat{H}}_{D}\psi =\left(c\hat{\sigma }\cdot \hat{p}+m_{0}c^{2}{\hat{\sigma }}_{z}\right)\psi =E\psi ,\;\psi =\left(\begin{array}{c} \psi_{1}\\ \psi_{2} \end{array}\right).$$

Here, ${\hat{H}}_{D}$ denotes the Dirac Hamiltionian, $\hat{\sigma }=\left({\hat{\sigma }}_{x},{\hat{\sigma }}_{y}\right)$, ${\hat{\sigma }}_{x,y,z}$ are the Pauli matrices, and $E=\hbar ck_{E}=\hbar ck\sqrt{1+\beta^{2}}$ is the energy of the particle, where *k* is the free-space wave vector and $\beta =\frac{m_{0}c}{\hbar k}$ is the ratio of the rest-energy momentum and free-space momentum. The NB was introduced in Ref. [38] for the ultrarelativistic, i.e., massless case $m_{0}=0$. It is characterized by the way the particle is confined to the billiard domain Ω without destroying the self-adjointness of the Hamiltonian. This is ensured by imposing along its boundary ∂Ω on the solutions of Equation (1) the BC that the normal component of the local current, which is given by the expectation value of the current operator $\hat{u}=\nabla_{p}{\hat{H}}_{D}=c\hat{\sigma }$, $u\left(r\right)=c\psi^{†}\hat{\sigma }\psi$, vanishes, yielding independently of the mass [38,42],

(2) $$\psi_{2}\left(t\right)=i\mu e^{i\alpha (t)}\psi_{1}\left(t\right).$$

Here, the boundary r(t)=[x(t),y(t)] or in the complex plane w(t)=x(t)+iy(t) is parameterized in terms of *t*, and μ=±1 determines the rotational direction of the flow at the boundary, where it is unidirectional [38]. We chose μ=1. The parameter is defined for rectangular billiards with side lengths 2a and 2b in a coordinate system, whose origin is at its center and with the *x*-axis parallel to the sides with lengths 2a where w(t)=t±ib,t∈[−a,a], while the *y*-axis is parallel to those with lengths 2b where w(t)=±a+it,t∈[−b,b]. Furthermore, α(t) is the angle of the outward-pointing normal vector n(t) at r(t) with respect to the *x*-axis. Another choice of parameter which is commonly used is the arc-length s∈[0,L] with L denoting the perimeter, which essentially corresponds to *t* plus the sum of the lengths of the sides that have been passed when moving along the boundary where we set s=0 at r=(0,−b). In a local coordinate system (n,s) which moves counterclockwise along the boundary (n=0,s) and whose coordinate axes are in the directions of the tangential vector t(s) to ∂Ω at r(s) and the normal vector n(s), respectively, the combination of the Dirac Equation (1) at the boundary and Equation (2) yields with $\left(\partial_{x}\pm i\partial_{y}\right)=e^{\pm i\alpha }\left(\partial_{n}\pm i\partial_{s}\right)$ BCs in terms of separate equations for the wave-function components [42],

(3) $$\begin{array}{cc} {\left.\left(\partial_{n}+i\partial_{s}\right)\psi_{1}\left(n,s\right)\right|}_{n\to 0^{-}} & =-kK^{-1}\psi_{1}\left(s\right),\\ {\left.\left(\partial_{n}-i\partial_{s}\right)\psi_{2}\left(n,s\right)\right|}_{n\to 0^{-}} & =kK\psi_{2}\left(s\right). \end{array}$$

Here, $K=\sqrt{\frac{1-\sin\theta_{\beta }}{1+\sin\theta_{\beta }}}$ with $\sin\theta_{\beta }=\frac{\beta }{\sqrt{1+\beta^{2}}}$. Note that when introducing

(4) $$\psi =\left(\begin{array}{c} \sqrt{\frac{1+\sin\theta_{\beta }}{2}}{\tilde{\psi }}_{1}\\ \sqrt{\frac{1-\sin\theta_{\beta }}{2}}{\tilde{\psi }}_{2} \end{array}\right),$$

the Dirac equation Equation (1) takes the form of that for massless neutrinos with modified BCs,

(5) $$\begin{array}{cc} \; & k\tilde{\psi }\left(r\right)+i\hat{\sigma }\cdot \nabla \tilde{\psi }\left(r\right)=0 \end{array}$$

(6) $$\begin{array}{cc} \; & K{\tilde{\psi }}_{2}\left(s\right)=ie^{i\alpha (s)}{\tilde{\psi }}_{1}\left(s\right). \end{array}$$

The nonrelativistic limit is reached when the energy is close to the rest energy, $E≃m_{0}c^{2}$ [43], that is, for sufficiently large β→∞, corresponding to $K≃\frac{1}{2\beta }\to 0$ and $\theta_{\beta }\to \pi /2$. Conversely, in the ultrarelativistic case $m_{0}=0$, they equal K=1 and $\theta_{\beta }=0$. The BC imposes a phase relation on the wave-function components $\psi_{1,2}\left(s\right)$ at ∂Ω and provides a quantization condition whose solutions are the eigenstates of the Hamiltonian ${\hat{H}}_{NB}$ associated with the NB. Alternative BCs for the confinement of relativistic particles to a bounded domain are proposed in [70,72] and based on the ‘MIT’ bag model [73].

Exact analytical solutions were derived for the equilateral triangle NB [65,70], the circle NB [38] and the ellipse NB [41] based on plane wave expansions. In [74,75,76,77,78], the eigenenergies of Dirac particles confined to a one- and a three-dimensional box were computed based on a plane-wave ansatz employing the MIT bag model. However, even though the BCs depend either on *x* or on *y*, a complete quantization of the rectangular NB with such an ansatz is not possible, because the *x* and *y* parts of the kinetic energy term do not commute [38]. A plane wave expansion

(7) $$\begin{array}{ccc} & & \psi_{1}\left(r\right)=\sum_{j}a_{j}\left(k\right)e^{ik_{j}\cdot r} \end{array}$$

(8) $$\begin{array}{ccc} & & \psi_{2}\left(r\right)=\sum_{j}e^{i\theta_{j}}a_{j}\left(k\right)e^{ik_{j}\cdot r} \end{array}$$

with $k_{j}=k\left(\cos\theta_{j},\sin\theta_{j}\right)$ and BC Equation (2) or, equivalently, Equation (3) yields only one-dimensional solutions corresponding to eigenmodes propagating parallel to the *x* or *y* axis, for which either $\theta_{j}=0,\pi$ or $\theta_{j}=\frac{\pi }{2},\frac{3\pi }{2}$. Therefore, we employed an extension of the boundary-integral equations (BIEs) derived in Ref. [38] for massless NBs to massive ones [42,79].

The derivation is based on Green’s theorem, which provides exact integral equations for the eigenvalues and the spinor components in the interior of the billiard in terms of those on the boundary. An advantage of the boundary integral approach is that the eigenvalue problem is reduced from a two-dimensional differential equation to a one-dimensional boundary integral. The BIE is given by

(9) $$\left(1-\sin\theta_{\beta }\right){\tilde{\psi }}_{1}^{*}\left(\phi^{\prime }\right)=\frac{ik}{4}{⨏}_{0}^{2\pi }\left|w^{\prime }\left(\phi \right)\right|d\phi Q_{1}\left(\phi^{\prime },\phi \right){\tilde{\psi }}_{1}^{*}\left(\phi \right)$$

where the integration variable ϕ is related to *s* by $ds=|w^{\prime }\left(\phi \right)|d\phi$ and

(10) $$\begin{array}{cc} Q_{1}\left(\phi^{\prime },\phi \right)= & \cos\theta_{\beta }\left[e^{i\left(\alpha \left(\phi^{\prime }\right)-\alpha \left(\phi \right)\right)}-1\right]H_{0}^{\left(1\right)}\left(k\rho \right)\\ + & \left\{\left[1-\sin\theta_{\beta }\right]e^{i\left(\xi \left(\phi ,\phi^{\prime }\right)-\alpha \left(\phi \right)\right)}+\left[1+\sin\theta_{\beta }\right]e^{-i\left(\xi \left(\phi ,\phi^{\prime }\right)-\alpha \left(\phi^{\prime }\right)\right)}\right\}H_{1}^{\left(1\right)}\left(k\rho \right), \end{array}$$

with

(11) $$e^{i\xi (\phi ,\phi^{\prime })}=\frac{w\left(\phi \right)-w\left(\phi^{\prime }\right)}{|w\left(\phi \right)-w\left(\phi^{\prime }\right)|},\;\rho \left(\phi ,\phi^{\prime }\right)=\left|w\left(\phi \right)-w\left(\phi^{\prime }\right)\right|.$$

Here, $H_{m}^{\left(1\right)}\left(k\rho \right)=J_{m}\left(k\rho \right)+iY_{m}\left(k\rho \right)$ is the Hankel function of the first kind of order *m*. At $\phi =\phi^{\prime }$, i.e., ρ=0, $H_{0}^{\left(1\right)}\left(k\rho \right)$ and $H_{1}^{\left(1\right)}\left(k\rho \right)$ have a logarithmic and a 1/ρ singularity. The integral over these singularities leads to the $\sin\theta_{\beta }$ term on the left-hand side [79]. Accordingly, an interval $\left[\phi^{\prime }-\delta \phi ,\phi^{\prime }+\delta \phi \right]$, where δϕ is arbitrarily small, is excluded from the integration range on the right-hand side. The corresponding BIE for ${\tilde{\psi }}_{2}^{*}\left(\phi^{\prime }\right)$ is obtained by employing in Equation (10) the BC in Equation (6) and those for the spinor components $\psi_{1,2}\left(\phi \right)$ are obtained with Equation (4). In the nonrelativistic limit $\sin\theta_{\beta }\to 1$, the left-hand side approaches zero, implying that the evaluation of the BIE becomes a numerical challenge. We would like to note that for $\sin\theta_{\beta }≃1$, the BIE in Equation (10) does not provide a suitable quantization procedure since the spinor components decouple, $\psi_{2}\left(r\right)$ becomes vanishingly small and the BC for $\psi_{1}\left(r\right)$ turns into Robin BCs, which become Dirichlet BCs for $\sin\theta_{\beta }=1$. For that reason, the BIEs are replaced in the nonrelativistic limit by one for its normal derivative [80].

We are interested in the spectral properties and properties of the wave functions of the associated symmetry-projected eigenstates of rectangular NBs which have mirror symmtries with respect to the *x* and *y* axes and a twofold rotational symmetry—for the square NB, (a=b) even a fourfold symmetry. Yet, in this respect, there is a crucial difference between QBs and NBs, which has its origin in the fact that the BCs for NBs connect the spinor components $\psi_{1}\left(s\right)$ and $\psi_{2}\left(s\right)$ at ∂Ω and may lead to distinct spectral properties, as outlined in [66,71,81]. The characteristics of NBs under a reflection or a rotation operator can be summarized as follows.

Applying on the coordinate vector an orthogonal transformation, $r^{\prime }=\hat{R}r$ corresponds to applying a unitary transformation $\hat{U}$ to the Dirac Hamiltonian ${\hat{H}}_{D}\left(r\right)$. If $\tilde{\psi }\left(r\right)$ is a solution of the Dirac equation in Equation (1), then the eigenfunction of the transformed Dirac Hamiltonian ${\hat{H}}_{D}\left(r^{\prime }\right)={\hat{U}}^{†}{\hat{H}}_{D}\hat{U}$ is given by $\bar{\psi }\left(r^{\prime }\right)={\hat{U}}^{†}\tilde{\psi }\left(r\right)$. The unitary operators for a mirror reflection ${\hat{R}}_{x}={\hat{\sigma }}_{z}$ at the *x*-axis or ${\hat{R}}_{y}=-{\hat{\sigma }}_{z}$ at the *y*-axis are ${\hat{U}}_{x}={\hat{\sigma }}_{x}$ or ${\hat{U}}_{y}=i{\hat{\sigma }}_{y}$, respectively. However, application of the reflection operator ${\hat{R}}_{X}$ with X=x or X=y on a spinor eigenfunction $\tilde{\psi }\left(r\right)$ of ${\hat{H}}_{NB}\left(r\right)$ does not yield an eigenfunction of ${\hat{H}}_{NB}\left(r^{\prime }\right)$ [82], because $\bar{\psi }\left(r^{\prime }={\hat{R}}_{X}r\right)$ does not fulfill the BC in Equation (2).

The unitary operator ${\hat{U}}_{M}$ corresponding to a counterclockwise rotation by $\frac{2\pi }{M}$, $r^{\prime }={\hat{R}}_{M}r$ with

(12) $${\hat{R}}_{M}=\left(\begin{array}{cc} \cos\left(\frac{2\pi }{M}\right) & -\sin\left(\frac{2\pi }{M}\right)\\ \sin\left(\frac{2\pi }{M}\right) & \;\cos\left(\frac{2\pi }{M}\right) \end{array}\right),$$

reads

(13) $${\hat{U}}_{M}=\left(\begin{array}{cc} e^{i\frac{\pi }{M}} & 0\\ 0 & e^{-i\frac{\pi }{M}} \end{array}\right).$$

The eigenfunctions of the transformed Dirac Hamiltonian are ${\bar{\psi }}_{M}^{T}\left(r^{\prime }\right)=[e^{-i\frac{\pi }{M}}{\tilde{\psi }}_{1}\left(r\right),$$e^{i\frac{\pi }{M}}{\tilde{\psi }}_{2}\left(r\right)]$. For NBs whose boundary has an *M*-fold rotational symmetry $w\left(s^{\prime }\right)=e^{i\frac{2\pi }{M}}w\left(s\right)$ and $e^{i\alpha (s^{\prime })}=e^{i\frac{2\pi }{M}}e^{i\alpha (s)}$, the BC in Equation (2) is fulfilled for $\bar{\psi }\left(r^{\prime }\right)$ if $\tilde{\psi }\left(r\right)$ is an eigenfunction of ${\hat{H}}_{NB}$. Thus, the Hamiltonian ${\hat{H}}_{NB}\left(r\right)$ can be brought to a block diagonal form according to the *M* one-dimensional irreducible representations labeled by l=0,…,M−1; that is, its eigenstates can be grouped into *M* subspaces defined by their transformation properties under a rotation by $\frac{2\pi }{M}$ [83,84,85,86], yielding the symmetry-projected eigenstates

(14) $$\psi_{1,2}^{\left(l\right)}\left({\hat{R}}_{M}^{-\lambda }r\right)=e^{i\lambda \frac{2l\pi }{M}}{\tilde{\psi }}_{1,2}^{\left(l\right)}\left(r\right),\lambda =0,\ldots M-1.$$

The wave-function components corresponding to l=0 are invariant under rotation by $\frac{2\pi }{M}$, whereas for l≠0, a rotation by 2π is needed to recover the original ones. The Dirac equation in Equation (1) and the BC in Equation (6) relate components of the spinor eigenfunction with different transformation properties under rotation by $\frac{2\pi }{M}$ [65,71]. Namely, if the first component belongs to the subspace labeled by *l*,

(15) $${\tilde{\psi }}_{1}^{\left(l\right)}\left(r^{\prime }\right)=e^{-il\frac{2\pi }{M}}{\tilde{\psi }}_{1}^{\left(l\right)}\left(r\right)$$

where $r^{\prime }={\hat{R}}_{M}r$, then inserting this property into the Dirac equation yields for the second one

(16) $${\tilde{\psi }}_{2}\left(r^{\prime }\right)=e^{-i\left(l-1\right)\frac{2\pi }{M}}{\tilde{\psi }}_{2}\left(r\right)\equiv {\tilde{\psi }}_{2}^{\left(l-1\right)}\left(r^{\prime }\right),$$

where l=−1 corresponds to l=M−1. Similarly, using the *M*-fold symmetry of the boundary of the NB, i.e., $e^{i\alpha (s^{\prime })}=e^{i\left(\alpha \left(s\right)+\frac{2\pi }{M}\right)}$ and inserting Equation (15) into the BC in Equation (6) implies that [65,71]

$${\tilde{\psi }}_{2}\left(s^{\prime }\right)=ie^{i\alpha (s^{\prime })}K^{-1}{\tilde{\psi }}_{1}^{\left(l\right)}\left(s^{\prime }\right)=e^{-i\left(l-1\right)\frac{2\pi }{M}}{\tilde{\psi }}_{2}\left(s\right)\equiv {\tilde{\psi }}_{2}^{\left(l-1\right)}\left(s^{\prime }\right).$$

Thus, the spinor components cannot be simultaneously rotationally invariant, implying that the spinor eigenfunctions cannot be rotationally invariant. This intermingling of symmetry properties has its origin in the additional spin degree of freedom [65,71].

For a billiard whose boundary has an *M*-fold rotational symmetry, the corresponding symmetry-reduced one is constructed by cutting it along the sides of a fundamental domain into sectors with inner angle $\phi_{0}=\frac{2\pi }{M}$. The corresponding symmetry-reduced QB and NB are obtained by imposing the same BCs along the cutlines as along the outer bondary, i.e., as for the full QB, respectively, NB. These are Dirichlet BCs for the QB. Accordingly, their eigenstates coincide with rotationally invariant ones of the full QB. For the corresponding symmetry-reduced NB, the BCs in Equation (2) are imposed along the cutlines on the outgoing current, which is in the opposite direction to the current in the full NB at one of the cutlines [66]. Thus, the intermingling of symmetries in Equations (15) and (16) of the wave-function components implies that the eigenstates of an NB with a rotationally symmetric boundary cannot be eigenstates of the corresponding symmetry-reduced NB. Accordingly, their spectral properties do not necessarily coincide as demonstrated for circle and ellipse sectors in [66]. In the following, we will refer to the symmetry class of the spinor component ${\tilde{\psi }}_{1}^{\left(l\right)}\left(r\right)$ when specifying the value for *l*.

We would like to remark that the symmetry-projected eigenfunctions of a QB with *M*-fold rotational symmetry are complex for l≠0,M/2, implying that they are not invariant under application of the time-reversal operator $\hat{T}$ [83]. Indeed, the eigenfunctions with symmetry class *l* are transformed by $\hat{T}$ into eigenstates with $\tilde{l}=M-l$. Time-reversal invariance of the QB implies that the corresponding eigenvalues are doubly degenerate. It was demonstrated in [83] that the spectral properties of the states with l≠0,M/2 typically show GUE statistics, whereas those of the states with l=0,M/2 are well described by the GOE if the shape of the QB generates chaotic classical dynamics. On the other hand, the Dirac Hamiltonian in Equation (1) itself does not commute with the time-reversal operator $\hat{T}$. Consequently, if the shape has no mirror symmetry, for all *l*, the eigenvalues of the corresponding NB are not degenerate, and the spectral properties of massless NBs typically coincide with those of the GUE.

In the present work, we investigate the spectral properties of symmetry-projected and symmetry-reduced rectangular NBs. To compute the eigenstates, we applied the BIE. It can be separated into individual BIEs for each symmetry class. To take into account the *M*-fold symmetry of the boundary w(ϕ) of an NB, we use the periodicity properties

(17) $$\begin{array}{ccc} w\left(\phi +\lambda \frac{2\pi }{M}\right) & = & e^{i\lambda \frac{2\pi }{M}}w\left(\phi \right), \end{array}$$

(18) $$\begin{array}{ccc} w^{\prime }\left(\phi +\lambda \frac{2\pi }{M}\right) & = & e^{i\lambda \frac{2\pi }{M}}w^{\prime }\left(\phi \right), \end{array}$$

(19) $$\begin{array}{ccc} e^{i\alpha \left(\phi +\lambda \frac{2\pi }{M}\right)} & = & e^{i\lambda \frac{2\pi }{M}}e^{i\alpha (\phi )}, \end{array}$$

with λ=0,1,2,…,M−1. Restricting the range of $\phi^{\prime }$ to one fundamental domain, $\phi \in [0,\frac{2\pi }{M})$, the BIEs are given as

(20) $${\tilde{\psi }}_{1}^{(l)*}\left(\phi^{\prime }\right)=\int_{0}^{\frac{2\pi }{M}}d\phi {\tilde{M}}^{\left(l\right)}\left(k;\phi ,\phi^{\prime }\right){\tilde{\psi }}_{1}^{(l)*}\left(\phi \right)$$

with l=0,1,2,…,M−1 and

(21) $${\tilde{M}}_{1}^{\left(l\right)}\left(k;\phi ,\phi^{\prime }\right)=\sum_{\lambda =0}^{M-1}e^{i\frac{2l\pi }{M}\lambda }M_{1,\lambda }\left(k;\phi ,\phi^{\prime }\right),$$

where

(22) $$M_{1,\lambda }\left(k;\phi ,\phi^{\prime }\right)=Q_{1}\left(k;\phi +\lambda \frac{2\pi }{M},\phi^{\prime }\right).$$

## 3. Tools Employed for the Analysis of Properties of the Eigenstates

We analyzed the spectral properties in terms of the nearest-neighbor spacing distribution P(s), the integrated nearest-neighbor spacing distribution I(s), the number variance $\Sigma^{2}\left(L\right)$ and the Dyson–Mehta statistic $\Delta_{3}\left(L\right)$, which provides a measure for the rigidity of a spectrum [2]. For this, the ordered eigenvalues $k_{m}=\sqrt{E_{m}}$ with $k_{1}\leq k_{2}\leq \ldots$ were unfolded to mean spacing unity by replacing them with the smooth part of the integrated spectral density, $\epsilon_{m}=N^{smooth}\left(k_{m}\right)$, which is given for QBs by Weyl’s formula [87] $N^{Weyl}\left(k_{m}\right)=\frac{A}{4\pi }k_{m}^{2}-\frac{L}{4\pi }k_{m}+C_{0}$, with A denoting the area of the billiard, whereas for massless NBs, the perimeter contribution cancels out [38]. Furthermore, we analyzed distributions of the ratios [88,89] of consecutive spacings between nearest neighbors, $r_{j}=\frac{\epsilon_{j+1}-\epsilon_{j}}{\epsilon_{j}-\epsilon_{j-1}}$, which are dimensionless so that unfolding is not needed [88,89,90].

We also computed Husimi functions which are defined in classical phase space [5,36]. The classical dynamics of a CB is determined by the shape of its boundary ∂Ω. Similarly, the eigenstates of the corresponding QB or NB are obtained by employing the BIEs originating from Green’s theorem and thus are fully determined by their values along ∂Ω. Therefore, an appropriate choice of the Poincaré surface of section (PSOS) is obtained by restricting the phase space to ∂Ω, which commonly is defined in terms of Poincaré–Birkhoff coordinates (s,p), where p=sinχ(s) with χ(s) denoting the angle of the particle trajectory with respect to the normal vector n(s). In Ref. [37], Poincaré–Husimi functions are defined for nonrelativistic QBs as the projection of the normal derivative of the associated wave function ψ(s) at the boundary onto a coherent state [37], which is localized at ∂Ω and periodic with period L,

$$H_{j}\left(s,p\right)=\frac{1}{2\pi k_{j}}\frac{1}{\int_{0}^{L}ds^{\prime }{\left|\partial_{n^{\prime }}\psi \left(s^{\prime }\right)\right|}^{2}}{\left|\int_{0}^{L}ds^{\prime }\partial_{n^{\prime }}\psi \left(s^{\prime }\right)C_{\left(s^{\prime },p\right)}^{\delta }\left(s^{\prime };k_{j}\right)\right|}^{2}.$$

Here, $n^{\prime }=n\left(s^{\prime }\right)$ and

(23) $$C_{\left(s,p\right)}^{\delta }\left(s^{\prime };k_{j}\right)={\left(\frac{k_{j}}{\pi \delta^{2}}\right)}^{1/4}\sum_{m=-\infty }^{\infty }\exp\left(ipk_{j}\left(s^{\prime }-s+mL\right)-\frac{k_{j}}{2\delta^{2}}{\left(s^{\prime }-s+mL\right)}^{2}\right).$$

The parameter δ controls the resolution of the Husimi plots. The Poincaré–Husimi function provides a probability density of the eigenstates on the Poincaré surface of section [37] and accordingly is commonly referred to as a quantum Poincaré surface of section. We computed Husimi functions for the NB by replacing ψ(s) by the symmetry-projected spinor eigenfunctions $\psi_{1,2}^{\left(l\right)}\left(s\right)$, where we employed Equation (3) to determine their normal derivatives.

We, furthermore, computed momentum distributions [35], i.e., the Fourier transform of the spinor eigenfunctions $\psi_{n}\left(r\right)$ from coordinate space (x,y) to momentum space $\left(q_{x},q_{y}\right)$,

(24) $$\Psi_{n}\left(q\right)={\;\int \int \;}_{\Omega }dre^{iqr}\psi_{n}\left(r\right).$$

They are localized on the energy shell, that is, at values $q=\sqrt{q_{x}^{2}+q_{y}^{2}}=k_{n}$, and provide information on the directions of the plane waves that form the eigenmode, which is especially useful when it is scarred along periodic orbits or localized.

## 4. Numerical Results for the Symmetry-Projected Eigenstates of Rectangular NBs

In this section, we present numerical results for the spectral properties and properties of the wave functions, Husimi functions and momentum distributions of the symmetry-projected eigenstates of rectangular NBs with different side-length ratios,

(25) $$\begin{array}{ccc} & & R_{1}\left(T_{1}\right):\;\frac{a}{b}=1\\ & & R_{2}\left(T_{2}\right):\;\frac{a}{b}=2\\ & & R_{3}\left(T_{3}\right):\;\frac{a}{b}=\frac{\sqrt{5}+1}{2}\\ & & R_{4}\left(T_{4}\right):\;\frac{a}{b}=\frac{\sqrt{2}+\sqrt{\pi }}{2}\\ & & R_{5}\left(T_{5}\right):\;\frac{a}{b}=\sqrt{3} \end{array}$$

and their symmetry-reduced counterparts, namely triangles $T_{i}$, that are obtained by cutting the corresponding rectangle $R_{i}$ along its diagonal. The triangles $T_{2},T_{3},T_{4}$ have in common that their angles have the structure $\left\{\alpha_{1}\pi ,\alpha_{2}\pi ,\frac{\pi }{2}\right\}$ with $\alpha_{1},\;\alpha_{2}$ irrational numbers. Furthermore, in [66], we considered a triangle, named $T_{5}$, which is obtained by cutting the equilataral triangle with side lengths 2 along a mirror axis into two halves. Its inner angles are $\left\{\frac{\pi }{2},\frac{\pi }{3},\frac{\pi }{6}\right\}$, implying that it is integrable like the $R_{1}$ and $T_{1}$ billiards. We computed 5000 eigenvalues for each symmetry class and NB. These studies were motivated by recent results which we obtained in Ref. [66] for the semi-circle NB, the quarter-ellipse NB for various values of its eccentricity, and the $T_{5}$ triangle NB [65]. For an ellipse with semimajor and semiminor axes lengths $a=\cosh\mu_{0}$ and $b=\sinh\mu_{0}$, respectively, the eccentricity is $\epsilon =\frac{1}{\cosh\mu_{0}}$. The classical dynamics of a particle in an ellipse CB and the semi- and quarter-ellipse CBs, which are obtained by cutting the ellipse along the semiminor and semimajor axes, respectively, is integrable because the product of the angular momenta with respect to the two focal points is a constant of motion. The eigenfunctions of the corresponding QBs are given by products of the radial and the periodic Mathieu functions [69,91,92,93,94], and the eigenvalues are the zeroes of the radial Mathieu function at the boundary. Their spectral properties coincide with those of Poissonian random numbers. The orbits of the ellipse CB and the eigenmodes of the ellipse QB can be separated into librational modes and rotational modes. With decreasing ϵ, the ellipse turns into a circle, and the librational modes turn into the diameter orbit, whereas with increasing eccentricity, the modes resemble those in the rectangular billiard. The eigenfunctions of the ellipse NB are given in terms of superpositions of products of the radial and periodic Mathieu functions [41]. It has a twofold symmetry so that the spinor components $\psi_{1}$ are either symmetric with respect to a rotation by π and the associated second component $\psi_{2}$ is antisymmetric, or vice versa.

We showed in [66] that the spectral properties of circle-sector NBs and quarter ellipse-sector NBs with small eccentricity, i.e., nearly circular shape, are well described by the GOE after the extraction of contributions from the diameter orbit in the former one and librational modes in the latter one that bounce back and forth parallel to the semiminor axis and have a vanishing support at the corners connecting straight and curved parts. This behavior was attributed to the intermingling of the symmetry properties associated with the rotational symmetry of the corresponding full NBs and the effects of these corners. Indeed, for the $T_{5}$ NB, the spectral properties are close to Poisson statistics [66], whereas we found good agreement with semi-Poissonian statistics for the symmetry-projected eigenstates of the equilateral triangle NB and QB [65].

**Figure 1:**
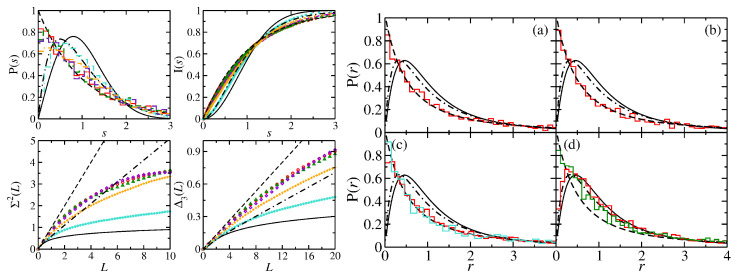
(**Left**) Spectral properties of the symmetry-projected eigenstates of the ellipse NB with ϵ=0.65 (red histogram and circles: l=0; green histogram and triangles: l=1), and the semi-ellipse NB with ϵ=0.65 (violet histogram and diamonds), ϵ=0.5 (orange histogram and plus) and ϵ=0.1 (turquoise histogram and stars). (**Right**) Ratio distributions for (**a**) the symmetry-projected eigenstates of the ellipse NB with l=0, (**b**) l=1, (**c**) all eigenvalues of the ellipse NB (red) and the corresponding ellipse QB (turquoise) and (**d**) the semi-ellipse NB for ϵ=0.5 (green) and ϵ=0.1 (red). They are compared to the GOE (black solid line), Poisson (black dashed line) and semi-Poisson (black dashed–dotted lines) statistics.

In Figure 1, we present some results for the semi-ellipse NB for different eccentricities. Shown are the spectral properties of the ellipse NB and its symmetry-projected eigenstates for ϵ=0.265 and for the semi-ellipse NBs with ϵ=0.1,0.5,0.65. Those of the symmetry-projected eigenstates are close to those of the semi-ellipse NB with ϵ=0.65, which is close to Poisson, whereas with decreasing ϵ, they approach semi-Poisson statistics, as is clearly visible in the ratio distributions which are shown in the right part of Figure 1.

**Figure 2:**
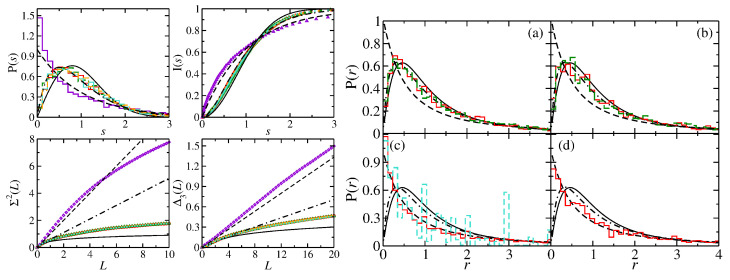
(**Left**) Spectral properties of the $R_{1}$, i.e., square NB (violet histogram and diamonds) and its symmetry-projected eigenstates (red histogram and circles: l=0; green histogram and triangles: l=1; orange histogram and plus: l=2; turquoise histogram and stars: l=3). (**Right**) Ratio distributions for (**a**) the symmetry-projected eigenstates of the $R_{1}$ NB with l=0 (red) and l=2 (green), (**b**) l=1 (red) and l=3 (green), (**c**) all eigenvalues of the square NB (red) and the corresponding square QB (turquoise) and (**d**) the $45^{\circ }$-triangle, i.e., $T_{1}$ NB (red). They are compared to the GOE (black solid line), Poisson (black dashed line) and semi-Poisson (black dashed–dotted lines) statistics.

In Figure 2, we show results for the square NB ($R_{1}$ in Equation (25)), its symmetry-projected eigenstates labeled by l=0,1,2,3 and the $45^{\circ }$ triangle ($T_{1}$ in Equation (25)).

The spectral properties of the symmetry-projected eigenstates agree well with semi-Poisson for the short-range correlations up to a certain number of mean spacings *L* for the long-range correlations. The size of the deviations is similar to that of a QB with integrable classical dynamics [20,24] for a similar number of eigenvalues, such as, e.g., the ellipse and circle QBs and NBs [41,66]. The overshooting of those for the square NB with respect to Poisson statistics originates from non-systematic, occasionally occurring degeneracies of eigenvalues associated with different symmetry classes, which have also been observed for the equilateral triangle NB in Ref. [65]. The ratio distribution of the $R_{1}$ QB is shown together with that of the $R_{1}$ NB in the right part of Figure 2 in (c). It exhibits the nongeneric behavior commonly observed for rectangular QBs with rational ratios of side lengths, whereas that of the NB agrees well with Poisson statistics.

**Figure 3:**
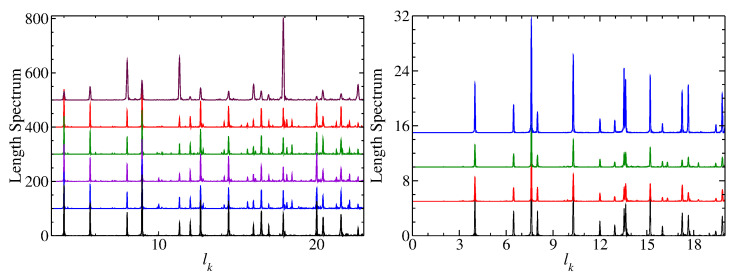
(**Left**) Comparison of the length spectra of, from bottom to top, the full square NB (black line), for l=0 (blue line), l=1 (violet line), l=2 (green), l=3 (red), and the square QB (maroon). (**Right**) Comparison of the length spectra of, from bottom to top, the rectangular $R_{3}$ NB (black line), for l=0 (red line), l=1 (green line), and the corresponding QB (blue).

In the left part of Figure 3, we compare length spectra, that is, the modulus of the Fourier transform $|{\tilde{\rho }}^{fluc}\left(l\right)|$ of the fluctuating part of the spectral density, $\rho^{fluc}\left(k\right)$, from wave number *k* to length *l* for the full square NB, the symmetry-projected ones and the $45^{\circ }$ triangle NB. The length spectra of the QB and NB exhibit peaks at the lengths of its periodic orbits. Generally, in the length spectra of NBs, peaks at lengths, which correspond to periodic orbits with an odd number of reflections, are missing [42]. Such orbits are absent in rectangular CBs. Those of the symmetry-projected eigenstates show additional peaks at lengths which correspond to pseudo-orbits, that is, orbits that are periodic in the fundamental domains but not in the full system [85,86].

**Figure 4:**
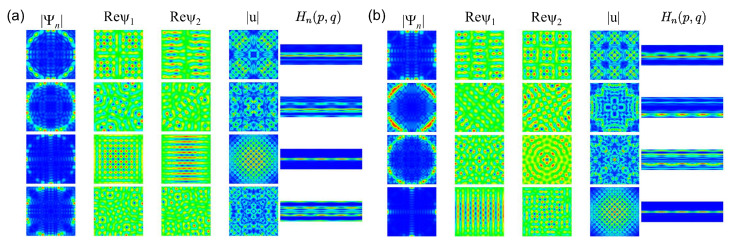
(**a**) From left to right, the momentum distribution in the $\left(q_{x},q_{y}\right)$ plane, the real parts of the spinor components $\psi_{1}\left(r\right)$ and $\psi_{2}\left(r\right)$ function in the (x,y) plane, the local current in the (x,y) plane and the Husimi functions in the Birkhoff coordinate plane (s,p), where s=0 at the center of the lower horizontal side and increases in counterclockwise direction, for the symmetry-projected eigenstates states of the square NB with l=0 and, from top to bottom, numbers n=49,51,68,75. (**b**) Same as left for l=1.

In Figure 4, we show examples for the momentum distributions, real parts of the spinor components $\psi_{1}\left(r\right)$ and $\psi_{2}\left(r\right)$, the local current $\left|u\left(r\right)\right|\propto \left|\left(ℜ\left[\psi_{1}^{*}\left(r\right)\psi_{2}\left(r\right)\right],ℑ\left[\psi_{1}^{*}\left(r\right)\psi_{2}\left(r\right)\right]\right)\right|$ and Husimi functions for the symmetry-projected eigenstates of the $R_{1}$ NB with l=0 (a) and l=1 (b). The wave-function patterns are invariant under rotation by $\frac{\pi }{2}$ for $\psi_{1}\left(r\right)$ and l=0, and for $\psi_{2}\left(r\right)$ and l=1. For the other components, they need to be rotated by 2π to recover the original patterns. The momentum distributions are peaked around values $\left(q_{x},q_{y}\right)=\left(\pm k_{x},\pm k_{y}\right)$ and $\left(q_{x},q_{y}\right)=\left(\pm k_{y},\pm k_{x}\right)$ along a circle, whose radius is defined by the eigenwavenumber $q=\sqrt{q_{x}^{2}+q_{y}^{2}}=k_{n}$ corresponding to the eigenstate number *n*. For n=68 and l=0 or for n=75 and l=1, it is peaked at $\left(\pm k_{n},0\right)$ and $\left(0,\pm k_{n}\right)$ and exhibit chessboard structures which resemble those of the square QB, except that their intensities decrease with the distance from the center of the square. Otherwise, the wave-function patterns are distinct and more complex. In addition, from the pattern structure of the local currents, we may conclude that the eigenfunctions are not given by simple superpositions of plane waves. Some of the Husimi functions are localized either in the upper or the lower part of the PSOS, indicating that the corresponding eigenmodes propagate in a preferred direction. This may be attributed to the fact that the BCs in Equation (2) lead to a unidirectionality of the local current along the boundary. The wave-function patterns are more complex than for the corresponding QB; however, they exhibit a regular structure, so that the GOE-like behavior of the spectral properties is not expected.

**Figure 5:**
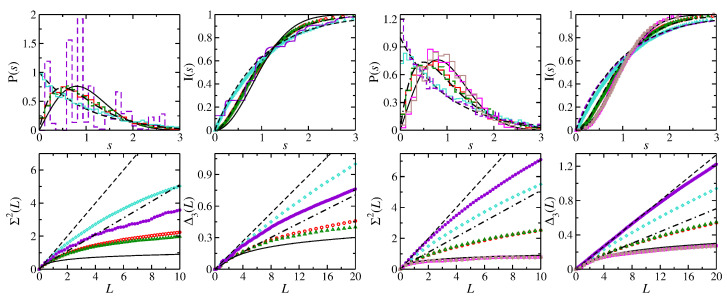
(**Left**) Spectral properties of the symmetry-projected eigenstates of the rectangular $R_{2}$ NB (red histogram and circles: l=0; green histogram and triangles: l=1), the $R_{2}$ QB (violet histogram, lines and dots), and the $R_{2}$ NB (turquoise histogram and diamonds). (**Right**) Spectral properties of the symmetry-projected eigenstates of the $R_{3}$ NB (red histogram and circles: l=0; green histogram and triangles: l=1), the $R_{3}$ QB (violet histogram, lines and dots), the $R_{3}$ NB (turquoise histogram and diamonds) and the triangular $T_{3}$ (magenta histogram and squares) and $T_{4}$ NB (brown histogram and dots). They are compared to the GOE (black solid line), Poisson (black dashed line) and semi-Poisson (black dashed–dotted lines) statistics.

**Figure 6:**
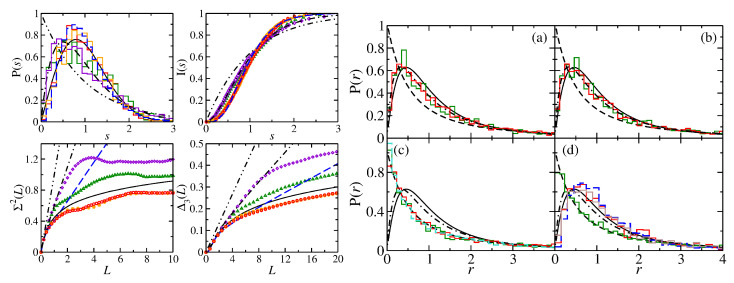
(**Left**) Spectral properties of the triangular $T_{3}$ NB (red histogram and circles) and $T_{3}$ QB (green histogram and triangles) and the triangular $T_{4}$ NB (orange histogram and dots) and $T_{4}$ QB (violet histogram and diamonds). They are compared to the GOE (black solid line), Poisson (black dashed–dotted–dotted line), semi-Poisson (black dashed–dotted lines) and quarter-Poisson (blue dashed lines) statistics. (**Right**) Ratio distributions for (**a**) the l=0 states of the $R_{2}$ (green histogram) and $R_{3}$ (red histogram) NBs, (**b**) same as (**a**) for the states with l=1, (**c**) the rectangular $R_{2}$ (green histogram) and $R_{3}$ (red histogram) NBs and for the rectangular $R_{3}$ QB (turquoise histogram), (**d**) the $T_{5}$ (green histogram), $T_{3}$ (red histogram) and $T_{4}$ (brown histogram) NBs. They are compared to the GOE (black solid line), Poisson (black dashed line), semi-Poisson (black dashed–dotted lines) and quarter-Poisson (blue dashed lines) statistics.

Similar results are obtained for the spectral properties of the rectangular $R_{2}$ and $R_{3}$ NBs and their symmetry-projected eigenstates, shown in the left and right part of Figure 5, respectively, and in the right part of Figure 6. The gaps observed in the nearest-neighbor spacing distribution of the $R_{2}$ QB are typical for rectangular QBs whose ratios of side lengths are no irrational numbers [95]. The long-range correlations, on the other hand, approach Poisson statistics with an increasing number of eigenvalues [24,96]. The spectral properties of the $R_{2}$ NB exhibit Poisson statistics, whereas those of its symmetry-projected eigenstates are well described by semi-Poisson statistics. In the right part of Figure 3, we show length spectra for the $R_{3}$ QB and NB and the symmetry-projected eigenstates of the $R_{3}$ NB. The length spectra of the QB and NB exhibit peaks at the same lengths, as there are only periodic orbits with an even number of reflections. The length spectra of the symmetry-projected eigenstates also exhibit peaks at the lengths of the classical periodic orbits and a few additional ones at the lengths of peudo-orbits.

**Figure 7:**
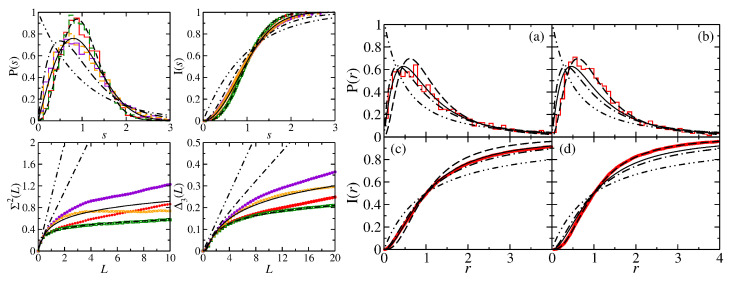
(**Left**) Spectral properties of the triangular $T_{2}$ QB and NB before ((violet histogram and stars) and (red histogram and dots)) and after extraction of contributions from orbits that lead to scarred wave functions ((orange dashed-line histogram and crosses) and (green histogram and squares)). They are compared to the GOE (black solid line), Poisson (black dashed–dotted–dotted line), semi-Poisson (black dashed–dotted lines) and GUE (black dashed lines) statistics. (**Right**) Ratio distributions P(r) (upper panels) and integrated ratio distributions I(r) (lower panels) for the $T_{2}$ QB (**a**,**c**) and NB (**b**,**d**). They are compared to the GOE (black solid line), Poisson (black dashed–dotted–dotted line), semi-Poisson (black dashed–dotted lines) and GUE (black dashed lines) statistics.

**Figure 8:**
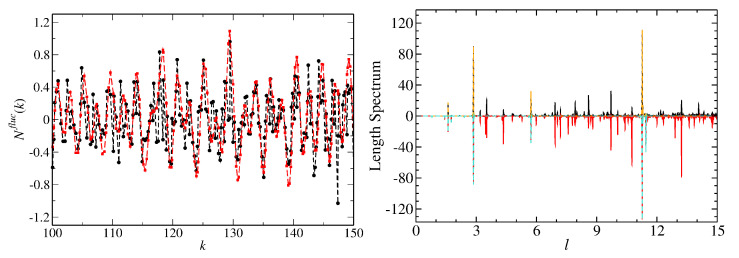
(**Left**) Fluctuating part of the integrated spectral density (black) and the contributions of bouncing-ball orbits that are extracted to obtain the results shown in Figure 7 (red) for the $T_{2}$ NB. (**Right**) Comparison of the length spectra of the $T_{2}$ NB (upper part) and the $T_{2}$ QB (lower part).

In the right part of Figure 5 and in Figure 6, we include the results for the triangular $T_{3}$ and $T_{4}$ NBs which are constructed by cutting the rectangular $R_{3}$ and $R_{4}$ NBs along the diagonals. Their spectral properties are close to GOE. We found out that they, actually, agree well with those of a quarter-Poissonian sequence which is obtained by taking from an ordered sequence of Possonian random numbers each fourth number or, equivalently, every second one from a sequence of semi-Poisson numbers, such as those of the corresponding symmetry-projected eigenvalue sequences. The spectral properties of the $T_{3}$ and $T_{4}$ QBs agree well with semi-Poisson statistics [34]. This is illustrated in the left part of Figure 6. Those of the $T_{5}$ NB coincide with Poissonian statistics [66]. Right triangles, that have only one angle which is rational with respect to π, have been studied in detail in Refs. [21,23,34,97,98]. It was shown that triangles, whose angles are all irrational with respect to π, exhibit GOE-like spectral properties, whereas those of right triangles are non-Poissonian but differ from GOE. Deviations from GOE were shown to originate from the presence of wave functions that are scarred along bouncing-ball orbits that exhibit a regular pattern, such as the periodic-orbit family consisting of orbits that are reflected perpendicular to the tilted side of the triangle and form a periodic-orbit channel (POC) whose maximum width extends from one diffractive corner to the other one (see below) [19,99,100]. Their contributions, in fact, can be extracted for irrational triangles by proceeding as, e.g., in [101], whereas for the $T_{3}$ and $T_{4}$ QBs and NBs, their number is too large. This explains the agreement with semi-Poisson statistics for the $T_{3}$ and $T_{4}$ QBs [34], which corresponds to a linear level repulsion and is a special case of intermediate statistics for which analytical expressions exist. These are shown as dashed–dotted lines, e.g., in Figure 6. For the corresponding NBs, the level repulsion is cubic [33]. After the extraction of contributions from bouncing-ball orbits, the spectral properties of the $T_{2}$ QB are close to GOE behavior, whereas those of the $T_{2}$ NB agree well with GUE statistics, as illustrated in Figure 7, implying that the effect of scarred wave functions is stronger for the QB than it is for the NB. Indeed, scarred wave functions occur more rarely for the $T_{2}$ NB than for the $T_{2}$ QB. The ratio distributions, shown in the right part of Figure 7, agree well with GOE and GUE for the $T_{2}$ QB and NB, respectively, even though contributions from periodic orbits that lead to scarred wave functions were not extracted, implying that it is insensitive to scarred states. Note that no unfolding is needed for the analysis of ratio distributions. The left part of Figure 8 shows the fluctuating part of the spectral density (black dots). The contributions from bouncing-ball orbits lead to slow oscillations. To determine them, we used the procedure introduced in [101], that is, we computed the Fourier transform of the fluctuating part of the spectral density, ${\tilde{\rho }}^{fluc}\left(l\right)$, and then the inverse Fourier transform over those parts that correspond to lengths of these orbits. The associated peaks are plotted as orange and turquoise dashed lines for the NB and QB, respectively, in the length spectrum shown in the right part of Figure 8. The resulting oscillating part of N(k), $N^{osc}\left(k\right)$, is shown as red dots connected by a red dashed line in the left part of Figure 8. To extract the contributions of this part to the spectral properties, the eigenvalues $k_{m}$ were unfolded by replacing them with the sum of the smooth and oscillating part of N(k), $\epsilon_{m}=N^{smooth}\left(k_{m}\right)+N^{osc}\left(k_{m}\right)$ [102].

**Figure 9:**
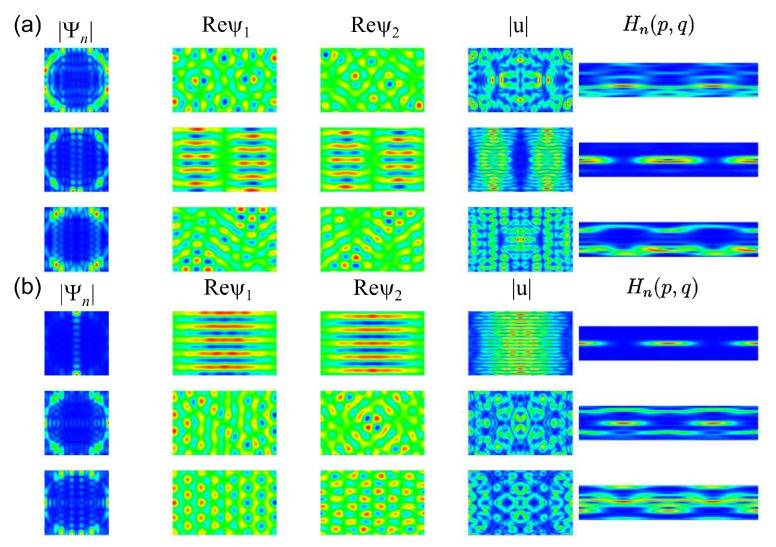
(**a**) Same as in Figure 4 for the symmetry-projected eigenstates of the rectangular $R_{3}$ NB with l=0 and, from top to bottom, numbers n=57–59. (**b**) Same as (**a**) for l=1.

**Figure 10:**
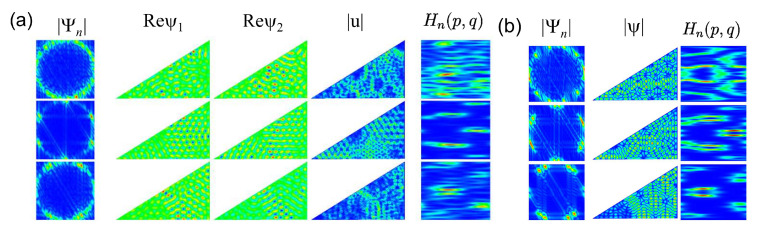
(**a**) Same as Figure 4 for the triangular $T_{3}$ NB with, from top to bottom, numbers n=255,288,264. Here, the Borkhoff coordinates are chosen such that s=0 at the left corner of the triangle, and it increases in the counterclockwise direction. (**b**) From left to right, momentum distributions, intensity distributions and Husimi functions for the corresponding QB with, from top to bottom, numbers n=266,259,267.

**Figure 11:**
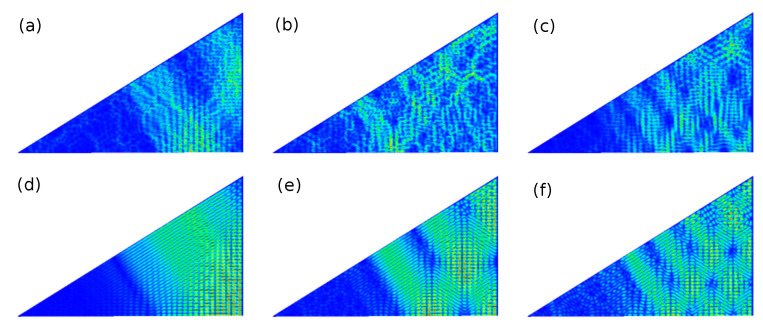
First row: Local current for the eigenstates of the $T_{3}$ NB for, from left to right, numbers n=1460 (**a**), 1453 (**b**), 1471 (**c**). Second row: intensity distribution for the eigenstates of the $T_{3}$ QB for, from left to right, numbers n=1459 (**d**), 1463 (**e**), 1469 (**f**).

In Figure 9, we show momentum distributions, real parts of the spinor components and Husimi functions for a few symmetry-projected eigenstates of the $R_{3}$ NB ((a): l=0; (b): l=1) and in Figure 10 for the corresponding right triangle $T_{3}$ NB (a) and $T_{3}$ QB (b). The momentum distributions are peaked at values $\left(q_{x},q_{y}\right)=\left(\pm k_{x},\pm k_{y}\right)$ along the circle defined by the eigenwavenumbers $k_{n}=\sqrt{q_{x}^{2}+q_{y}^{2}}$. In the example shown for l=1 and n=57, q takes the values $\left(q_{x},q_{y}\right)=\left(\pm k_{n},0\right)$. This case, in fact, can be derived based on a plane-wave ansatz, since the propagation of the eigenmodes is one-dimensional [77]. The wave-function patterns shown for l=0 in the second row are similar to corresponding ones in the $R_{3}$ QB. In these examples, the Husimi functions are localized along the p=0 axis. For the other cases, they again exhibit a preferred direction of propagation, but they are distributed over the (s,p) plane. Scarred wave functions of the type presented in [23] are observed in the corresponding symmetry-reduced billiard, i.e., the $T_{3}$ QB and NB. The examples shown in Figure 10 correspond to relatively low-lying states. Here, we chose for the QB eigenstates for which the wave functions and Husimi functions exhibit similar patterns to those of the NB. We show them because for higher excitations, the wave function and local current patterns become complex and are no longer discernible. Still, some of them exhibit scarred eigenstates, such as those shown in the third row. In Figure 11, we show examples of scarred wave functions for higher-lying eigenstates of the $T_{3}$ NB (upper row) and similar ones for the $T_{3}$ QB (lower row). They are scarred along bouncing ball orbits from the family of periodic orbits that hit the tilted side perpendicularly and are reflected with constant angles from the sides to the left and right of the $\frac{\pi }{2}$ corner.

## 5. Conclusions

We analyzed the properties of symmetry-projected eigenstates of rectangular NBs whose side lengths are either commensurable or incommensurable. Independently of the choice of the ratio of the side lengths, we find very good agreement of the spectral properties of the symmetry-projected eigenstates with semi-Poisson statistics. In distinction to the eigenvalues of rectangular QBs, those of the corresponding NBs are non-degenerate and exhibit Poisson statistics. The fact that the complete spectra of rectangular NBs exhibit Poisson statistics—whereas after their separation into two (or four) sequences of eigenvalues corresponding to the symmetry-projected eigenstates, the spectral properties follow semi-Poisson statistics—indicates that rectangular NBs behave like typical quantum systems with an integrable counterpart, whose eigenstates are non-degenerate and have alternating symmetry properties with increasing state number. Note that this is not strictly true, because when two eigenvalues come close to each other, then the ordering of the symmetry classes may change. Generally, Poisson statistics is expected for the symmetry-projected states if they are uncorrelated, as is the case for the corresponding nonrelativistic QB; however, in the ultrarelativistic case, they are linked through the different symmetry properties of the spinor components. Namely, if the first component belongs to class *l*, then the second one belongs to class (l−1). Furthermore, we found out that the eigenvalues of triangular NBs, which are constructed by cutting the rectangular NB with incommensurable side lengths along the diagonal and follow semi-Poisson statistics in the nonrelativistic limit, exhibit a cubic-level repulsion and spectral properties that agree well with those of random numbers. These random numbers are composed of every fourth number from a Poisson sequence or every second one from a semi-Poisson one. That is, we observe a hierarchy, induced by the variation of the BCs along the diagonal of the $R_{3}$ and $R_{4}$ NBs, in the spectral properties in the sense that they follow Poisson statistics for the full rectangle NB, semi-Poisson statistics for the symmetry-projected eigenstates and corresponding triangle QB and quarter-Poisson statistics for the symmetry-reduced NB, which would correspond to taking every second level from successive eigenvalue sequences. For the $T_{2}$ QB and NB, we find good agreement with GOE and GUE statistics, respectively, after the extraction of contributions from bouncing-ball orbits, that is, from eigenstates whose wave functions are scarred along these orbits. An important difference between a QB and the corresponding NB is that the associated Hamiltonian preserves and violates time-reversal invariance, respectively. Thus, the results for the $T_{2}$ NB are not surprising, and accordingly, we may deduce from our results for the $T_{3}$ and $T_{4}$ QBs and NBs that when inducing time-reversal invariance violation in a QB which exhibits semi-Poisson statistics, it will yield quarter-Poisson statistics. We chose these triangles because their spectral properties are particular and only considered the ultrarelativistic and the nonrelativistic limits. The transition region was investigated in detail in Refs. [65,71] for the symmetry-projected eigenstates of NBs whose boundaries have a threefold rotational symmetry. The results confirm that, as was shown in [42], with increasing mass, the spectral properties approach those of the nonrelativistic QB.

## Data Availability

All data are contained within this article.
